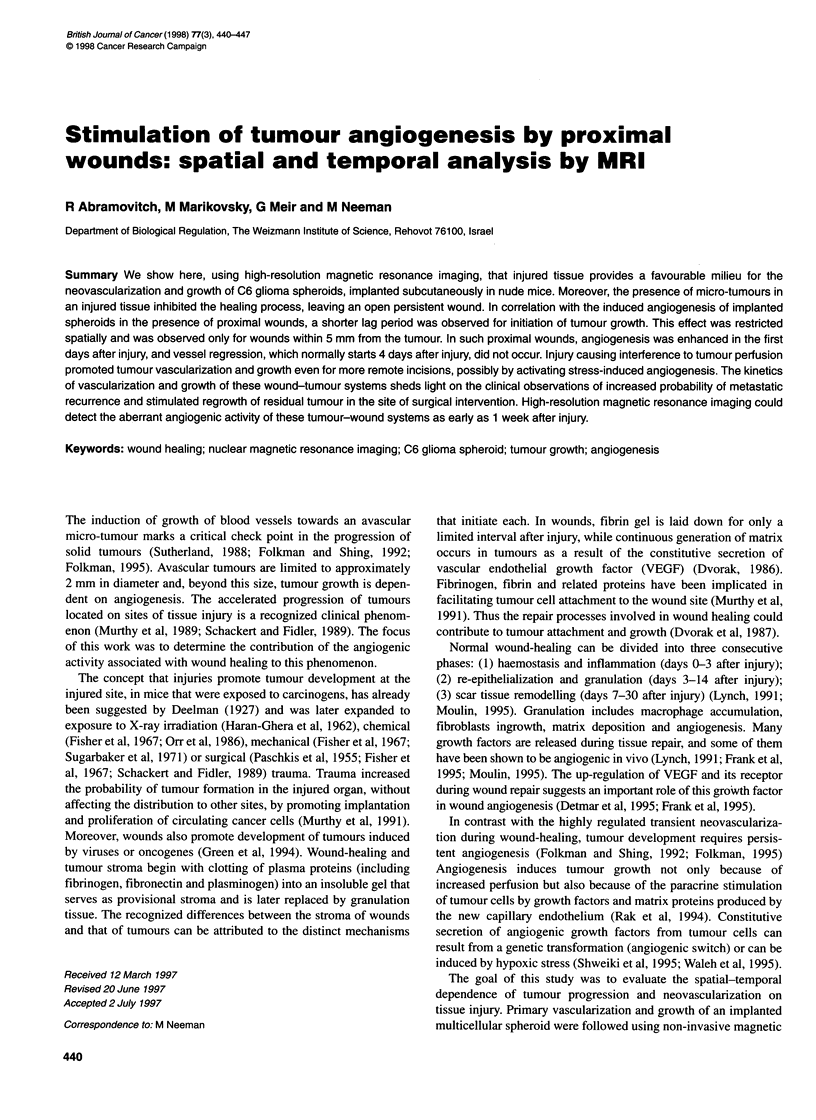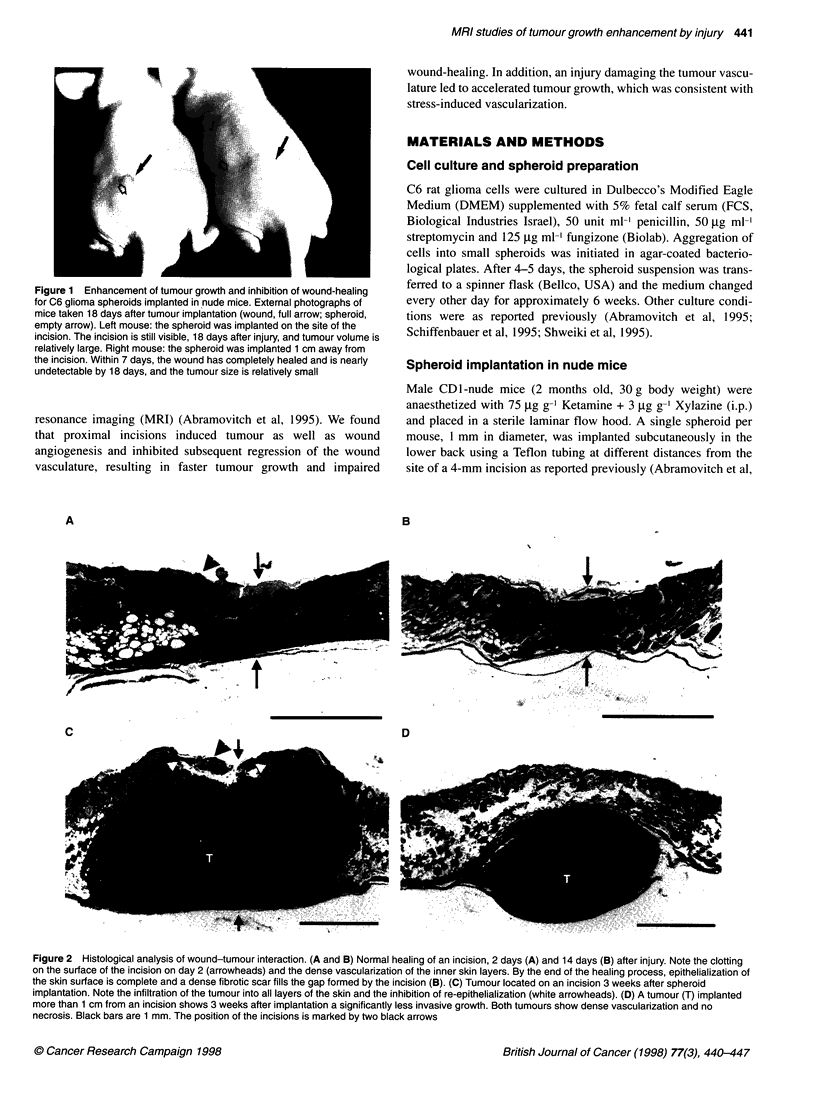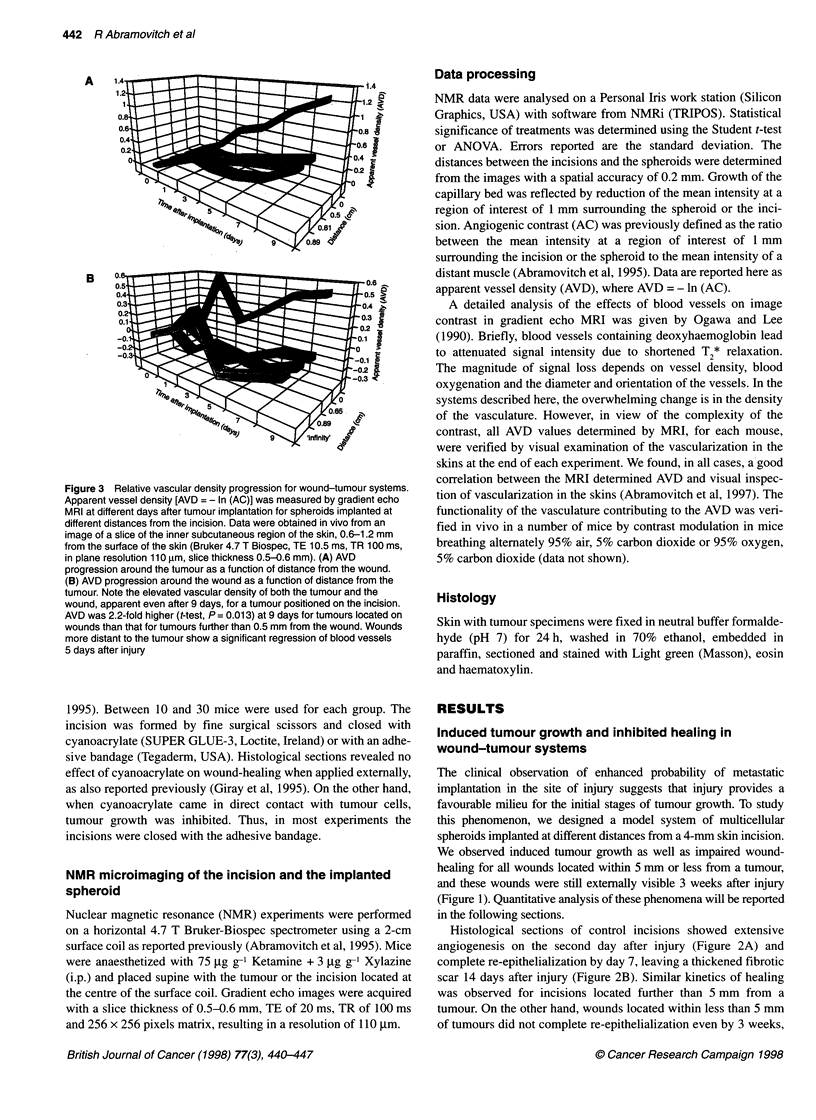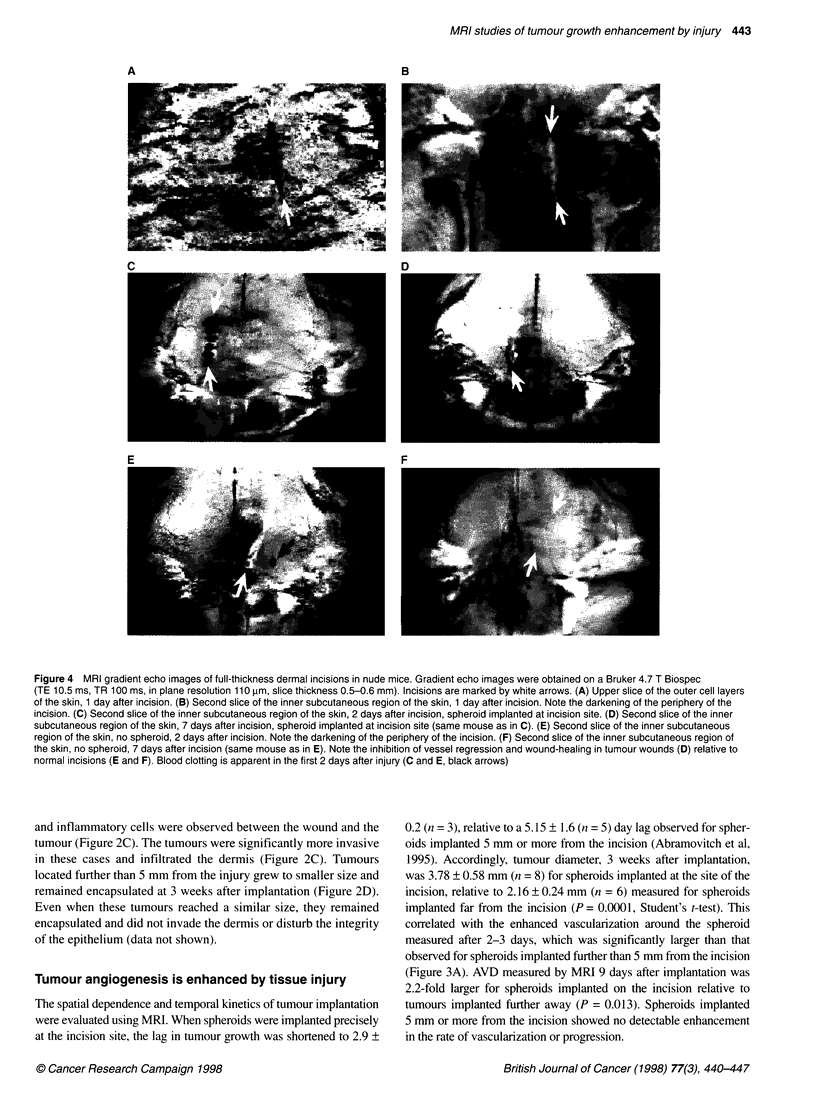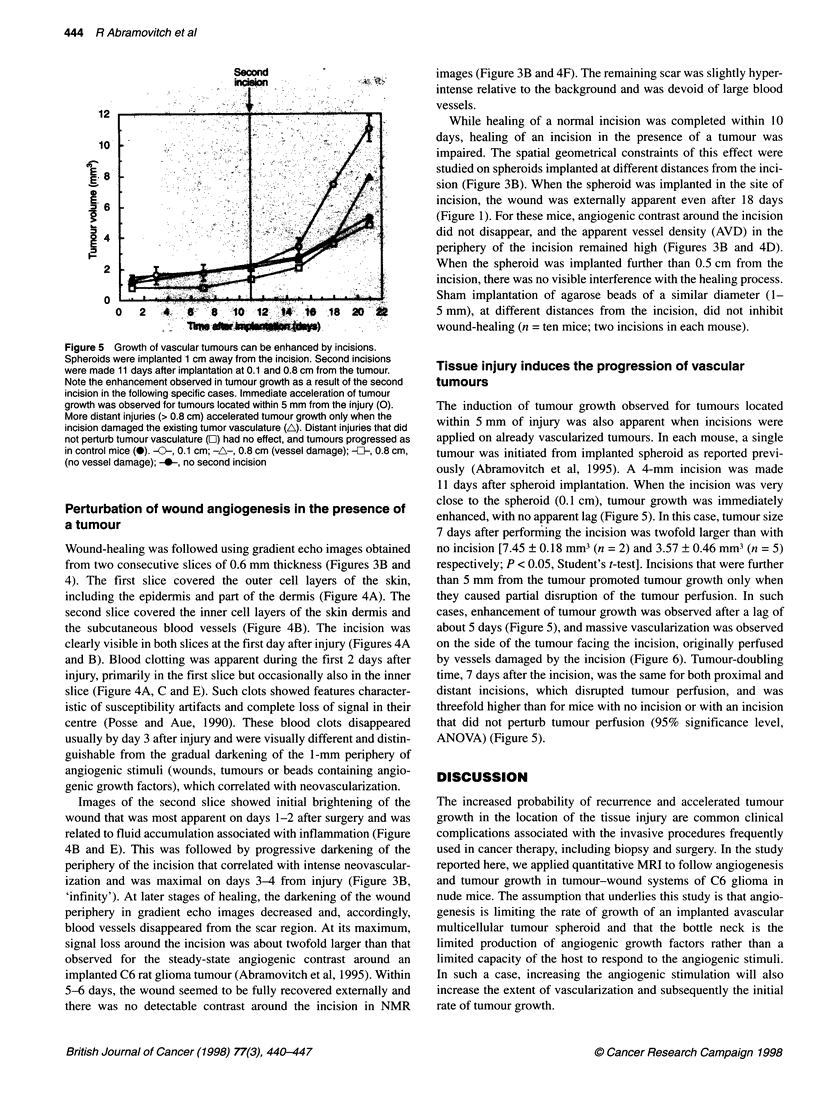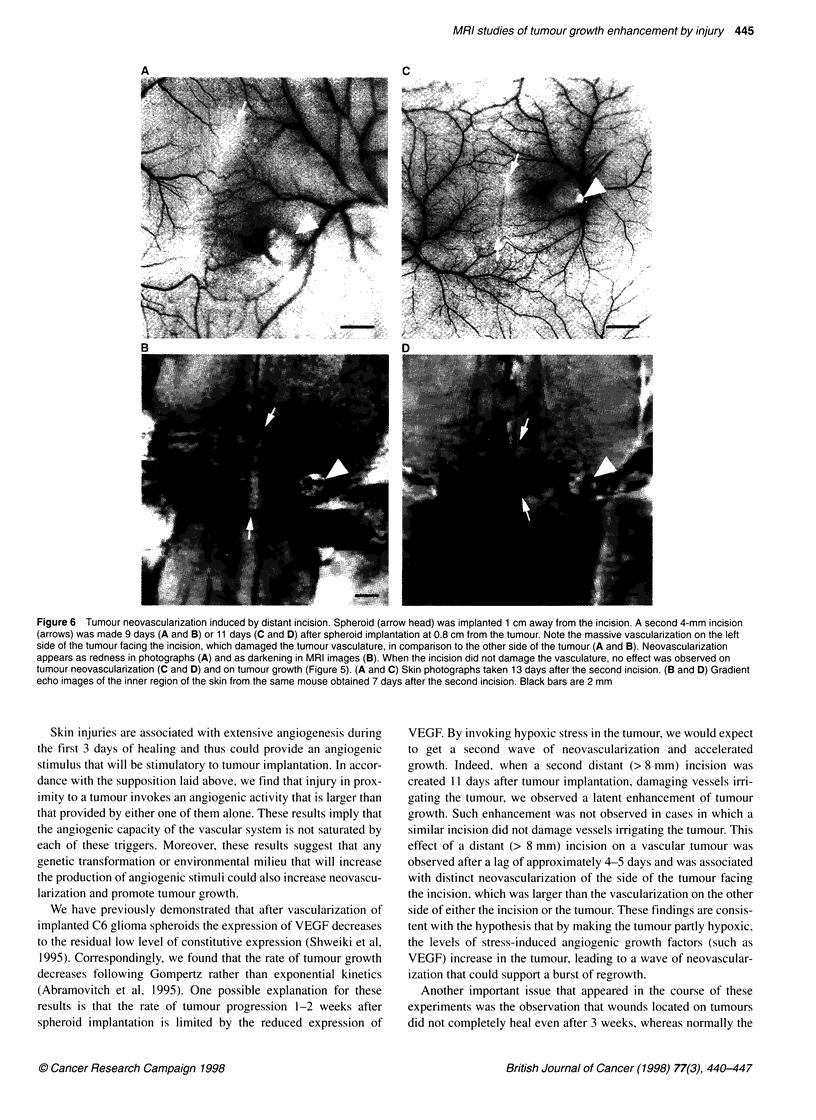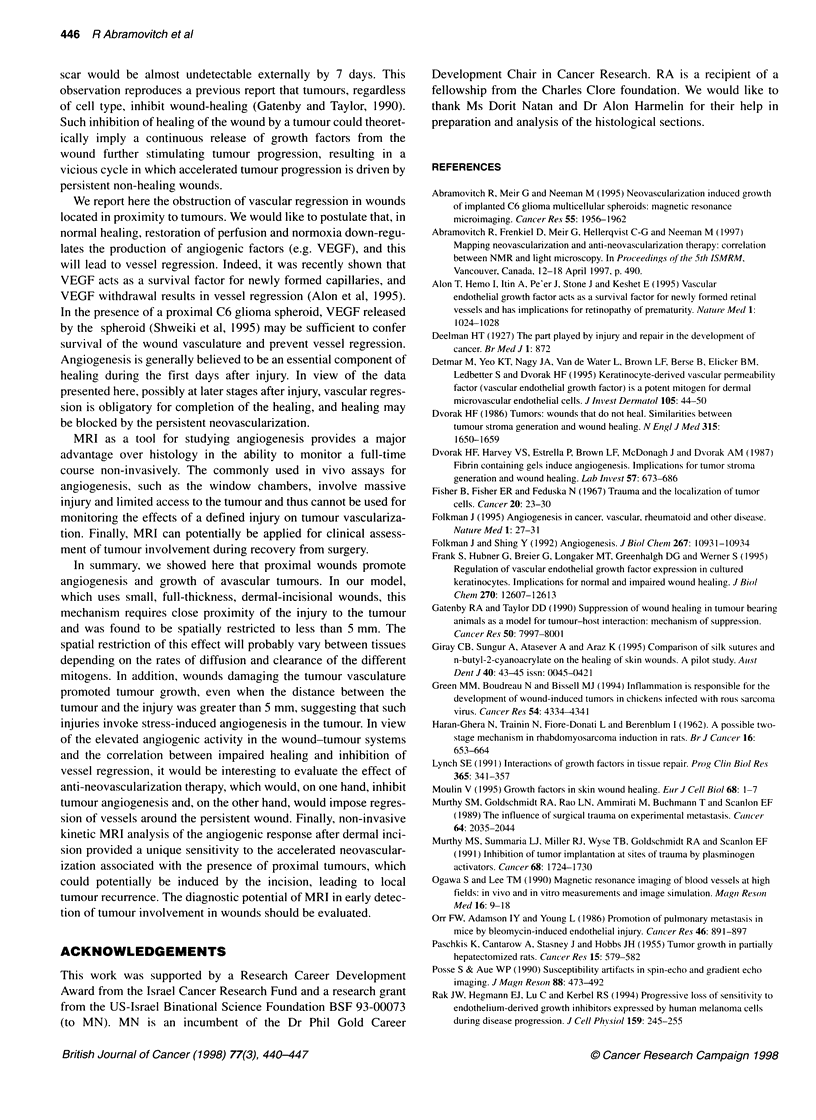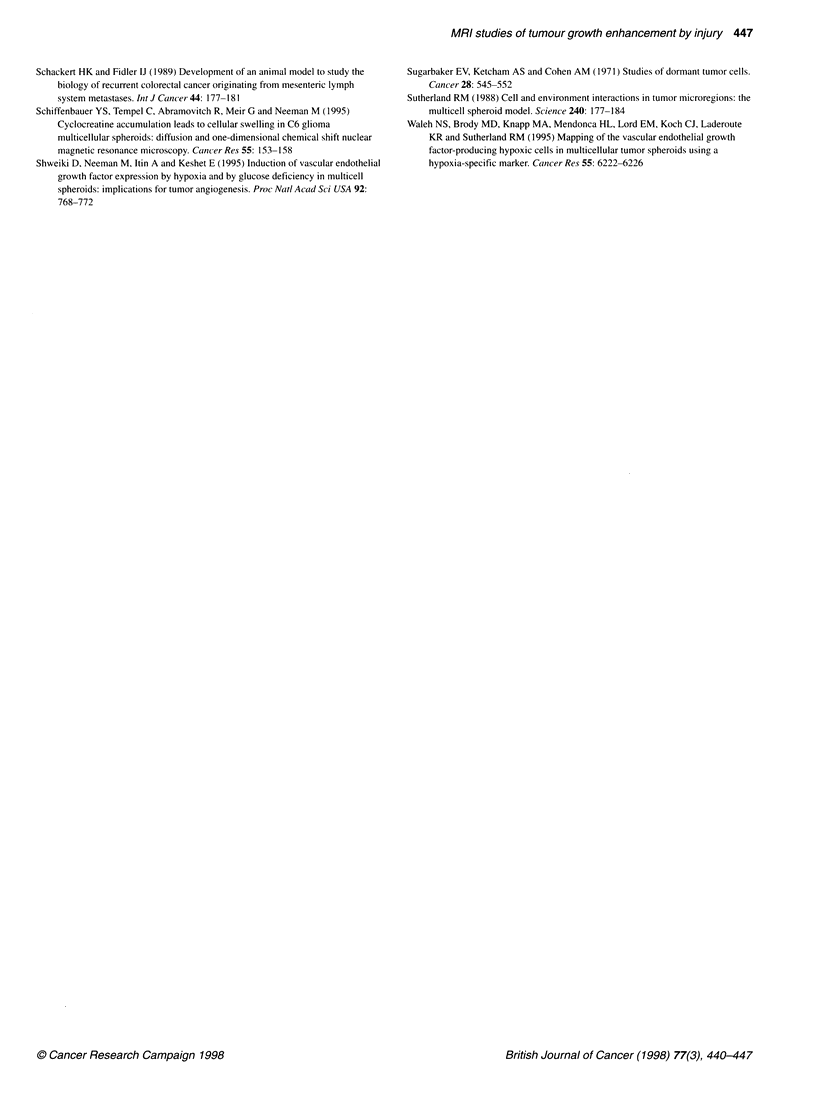# Stimulation of tumour angiogenesis by proximal wounds: spatial and temporal analysis by MRI.

**DOI:** 10.1038/bjc.1998.70

**Published:** 1998

**Authors:** R. Abramovitch, M. Marikovsky, G. Meir, M. Neeman

**Affiliations:** Department of Biological Regulation, The Weizmann Institute of Science, Rehovot, Israel.

## Abstract

**Images:**


British Journal of Cancer (1998) 77(3), 440–447
© 1998 Cancer Research Campaign

### Stimulation of tumour angiogenesis by proximal wounds: spatial and temporal analysis by MRI

**R Abramovitch, M Marikovsky, G Meir and M Neeman**

Department of Biological Regulation, The Weizmann Institute of Science, Rehovot 76100, Israel

**Summary** We show here, using high-resolution magnetic resonance imaging, that injured tissue provides a favourable milieu for the neovascularization and growth of C6 glioma spheroids, implanted subcutaneously in nude mice. Moreover, the presence of micro-tumours in an injured tissue inhibited the healing process, leaving an open persistent wound. In correlation with the induced angiogenesis of implanted spheroids in the presence of proximal wounds, a shorter lag period was observed for initiation of tumour growth. This effect was restricted spatially and was observed only for wounds within 5 mm from the tumour. In such proximal wounds, angiogenesis was enhanced in the first days after injury, and vessel regression, which normally starts 4 days after injury, did not occur. Injury causing interference to tumour perfusion promoted tumour vascularization and growth even for more remote incisions, possibly by activating stress-induced angiogenesis. The kinetics of vascularization and growth of these wound–tumour systems sheds light on the clinical observations of increased probability of metastatic recurrence and stimulated regrowth of residual tumour in the site of surgical intervention. High-resolution magnetic resonance imaging could detect the aberrant angiogenic activity of these tumour–wound systems as early as 1 week after injury.

**Keywords:** wound healing; nuclear magnetic resonance imaging; C6 glioma spheroid; tumour growth; angiogenesis

The induction of growth of blood vessels towards an avascular micro-tumour marks a critical check point in the progression of solid tumours (Sutherland, 1988; Folkman and Shing, 1992; Folkman, 1995). Avascular tumours are limited to approximately 2 mm in diameter and, beyond this size, tumour growth is dependent on angiogenesis. The accelerated progression of tumours located on sites of tissue injury is a recognized clinical phenomenon (Murthy et al, 1989; Schackert and Fidler, 1989). The focus of this work was to determine the contribution of the angiogenic activity associated with wound healing to this phenomenon.

The concept that injuries promote tumour development at the injured site, in mice that were exposed to carcinogens, has already been suggested by Deelman (1927) and was later expanded to exposure to X-ray irradiation (Haran-Ghera et al, 1962), chemical (Fisher et al, 1967; Orr et al, 1986), mechanical (Fisher et al, 1967; Sugarbaker et al, 1971) or surgical (Paschkis et al, 1955; Fisher et al, 1967; Schackert and Fidler, 1989) trauma. Trauma increased the probability of tumour formation in the injured organ, without affecting the distribution to other sites, by promoting implantation and proliferation of circulating cancer cells (Murthy et al, 1991). Moreover, wounds also promote development of tumours induced by viruses or oncogenes (Green et al, 1994). Wound-healing and tumour stroma begin with clotting of plasma proteins (including fibrinogen, fibronectin and plasminogen) into an insoluble gel that serves as provisional stroma and is later replaced by granulation tissue. The recognized differences between the stroma of wounds and that of tumours can be attributed to the distinct mechanisms that initiate each. In wounds, fibrin gel is laid down for only a limited interval after injury, while continuous generation of matrix occurs in tumours as a result of the constitutive secretion of vascular endothelial growth factor (VEGF) (Dvorak, 1986). Fibrinogen, fibrin and related proteins have been implicated in facilitating tumour cell attachment to the wound site (Murthy et al, 1991). Thus the repair processes involved in wound healing could contribute to tumour attachment and growth (Dvorak et al, 1987).

*Received 12 March 1997*
*Revised 20 June 1997*
*Accepted 2 July 1997*

*Correspondence to:* M Neeman

Normal wound-healing can be divided into three consecutive phases: (1) haemostasis and inflammation (days 0–3 after injury); (2) re-epithelialization and granulation (days 3–14 after injury); (3) scar tissue remodelling (days 7–30 after injury) (Lynch, 1991; Moulin, 1995). Granulation includes macrophage accumulation, fibroblasts ingrowth, matrix deposition and angiogenesis. Many growth factors are released during tissue repair, and some of them have been shown to be angiogenic in vivo (Lynch, 1991; Frank et al, 1995; Moulin, 1995). The up-regulation of VEGF and its receptor during wound repair suggests an important role of this growth factor in wound angiogenesis (Detmar et al, 1995; Frank et al, 1995).

In contrast with the highly regulated transient neovascularization during wound-healing, tumour development requires persistent angiogenesis (Folkman and Shing, 1992; Folkman, 1995) Angiogenesis induces tumour growth not only because of increased perfusion but also because of the paracrine stimulation of tumour cells by growth factors and matrix proteins produced by the new capillary endothelium (Rak et al, 1994). Constitutive secretion of angiogenic growth factors from tumour cells can result from a genetic transformation (angiogenic switch) or can be induced by hypoxic stress (Shweiki et al, 1995; Waleh et al, 1995).

The goal of this study was to evaluate the spatial–temporal dependence of tumour progression and neovascularization on tissue injury. Primary vascularization and growth of an implanted multicellular spheroid were followed using non-invasive magnetic

**Figure 1** Enhancement of tumour growth and inhibition of wound-healing for C6 glioma spheroids implanted in nude mice. External photographs of mice taken 18 days after tumour implantation (wound, full arrow; spheroid, empty arrow). Left mouse: the spheroid was implanted on the site of the incision. The incision is still visible, 18 days after injury, and tumour volume is relatively large. Right mouse: the spheroid was implanted 1 cm away from the incision. Within 7 days, the wound has completely healed and is nearly undetectable by 18 days, and the tumour size is relatively small resonance imaging (MRI) (Abramovitch et al, 1995). We found that proximal incisions induced tumour as well as wound angiogenesis and inhibited subsequent regression of the wound vasculature, resulting in faster tumour growth and impaired wound-healing. In addition, an injury damaging the tumour vasculature led to accelerated tumour growth, which was consistent with stress-induced vascularization.

### MATERIALS AND METHODS

### Cell culture and spheroid preparation

C6 rat glioma cells were cultured in Dulbecco's Modified Eagle Medium (DMEM) supplemented with 5% fetal calf serum (FCS, Biological Industries Israel), 50 unit $ml^{-1}$ penicillin, 50 $\mu g\ ml^{-1}$ streptomycin and 125 $\mu g\ ml^{-1}$ fungizone (Biolab). Aggregation of cells into small spheroids was initiated in agar-coated bacteriological plates. After 4–5 days, the spheroid suspension was transferred to a spinner flask (Bellco, USA) and the medium changed every other day for approximately 6 weeks. Other culture conditions were as reported previously (Abramovitch et al, 1995; Schiffenbauer et al, 1995; Shweiki et al, 1995).

### Spheroid implantation in nude mice

Male CD1-nude mice (2 months old, 30 g body weight) were anaesthetized with 75 $\mu g\ g^{-1}$ Ketamine + 3 $\mu g\ g^{-1}$ Xylazine (i.p.) and placed in a sterile laminar flow hood. A single spheroid per mouse, 1 mm in diameter, was implanted subcutaneously in the lower back using a Teflon tubing at different distances from the site of a 4-mm incision as reported previously (Abramovitch et al,

**Figure 2** Histological analysis of wound–tumour interaction. (**A** and **B**) Normal healing of an incision, 2 days (**A**) and 14 days (**B**) after injury. Note the clotting on the surface of the incision on day 2 (arrowheads) and the dense vascularization of the inner skin layers. By the end of the healing process, epithelialization of the skin surface is complete and a dense fibrotic scar fills the gap formed by the incision (**B**). (**C**) Tumour located on an incision 3 weeks after spheroid implantation. Note the infiltration of the tumour into all layers of the skin and the inhibition of re-epithelialization (white arrowheads). (**D**) A tumour (T) implanted more than 1 cm from an incision shows 3 weeks after implantation a significantly less invasive growth. Both tumours show dense vascularization and no necrosis. Black bars are 1 mm. The position of the incisions is marked by two black arrows

**Figure 3** Relative vascular density progression for wound–tumour systems. Apparent vessel density [AVD = – ln (AC)] was measured by gradient echo MRI at different days after tumour implantation for spheroids implanted at different distances from the incision. Data were obtained in vivo from an image of a slice of the inner subcutaneous region of the skin, 0.6–1.2 mm from the surface of the skin (Bruker 4.7 T Biospec, TE 10.5 ms, TR 100 ms, in plane resolution 110 μm, slice thickness 0.5–0.6 mm). (**A**) AVD progression around the tumour as a function of distance from the wound. (**B**) AVD progression around the wound as a function of distance from the tumour. Note the elevated vascular density of both the tumour and the wound, apparent even after 9 days, for a tumour positioned on the incision. AVD was 2.2-fold higher (*t*-test, $P = 0.013$) at 9 days for tumours located on wounds than that for tumours further than 0.5 mm from the wound. Wounds more distant to the tumour show a significant regression of blood vessels 5 days after injury

1995). Between 10 and 30 mice were used for each group. The incision was formed by fine surgical scissors and closed with cyanoacrylate (SUPER GLUE-3, Loctite, Ireland) or with an adhesive bandage (Tegaderm, USA). Histological sections revealed no effect of cyanoacrylate on wound-healing when applied externally, as also reported previously (Giray et al, 1995). On the other hand, when cyanoacrylate came in direct contact with tumour cells, tumour growth was inhibited. Thus, in most experiments the incisions were closed with the adhesive bandage.

### NMR microimaging of the incision and the implanted spheroid

Nuclear magnetic resonance (NMR) experiments were performed on a horizontal 4.7 T Bruker-Biospec spectrometer using a 2-cm surface coil as reported previously (Abramovitch et al, 1995). Mice were anaesthetized with 75 μg $g^{-1}$ Ketamine + 3 μg $g^{-1}$ Xylazine (i.p.) and placed supine with the tumour or the incision located at the centre of the surface coil. Gradient echo images were acquired with a slice thickness of 0.5–0.6 mm, TE of 20 ms, TR of 100 ms and 256 × 256 pixels matrix, resulting in a resolution of 110 μm.

### Data processing

NMR data were analysed on a Personal Iris work station (Silicon Graphics, USA) with software from NMRi (TRIPOS). Statistical significance of treatments was determined using the Student *t*-test or ANOVA. Errors reported are the standard deviation. The distances between the incisions and the spheroids were determined from the images with a spatial accuracy of 0.2 mm. Growth of the capillary bed was reflected by reduction of the mean intensity at a region of interest of 1 mm surrounding the spheroid or the incision. Angiogenic contrast (AC) was previously defined as the ratio between the mean intensity at a region of interest of 1 mm surrounding the incision or the spheroid to the mean intensity of a distant muscle (Abramovitch et al, 1995). Data are reported here as apparent vessel density (AVD), where AVD = – ln (AC).

A detailed analysis of the effects of blood vessels on image contrast in gradient echo MRI was given by Ogawa and Lee (1990). Briefly, blood vessels containing deoxyhaemoglobin lead to attenuated signal intensity due to shortened $T_2$* relaxation. The magnitude of signal loss depends on vessel density, blood oxygenation and the diameter and orientation of the vessels. In the systems described here, the overwhelming change is in the density of the vasculature. However, in view of the complexity of the contrast, all AVD values determined by MRI, for each mouse, were verified by visual examination of the vascularization in the skins at the end of each experiment. We found, in all cases, a good correlation between the MRI determined AVD and visual inspection of vascularization in the skins (Abramovitch et al, 1997). The functionality of the vasculature contributing to the AVD was verified in vivo in a number of mice by contrast modulation in mice breathing alternately 95% air, 5% carbon dioxide or 95% oxygen, 5% carbon dioxide (data not shown).

### Histology

Skin with tumour specimens were fixed in neutral buffer formaldehyde (pH 7) for 24 h, washed in 70% ethanol, embedded in paraffin, sectioned and stained with Light green (Masson), eosin and haematoxylin.

### RESULTS

### Induced tumour growth and inhibited healing in wound–tumour systems

The clinical observation of enhanced probability of metastatic implantation in the site of injury suggests that injury provides a favourable milieu for the initial stages of tumour growth. To study this phenomenon, we designed a model system of multicellular spheroids implanted at different distances from a 4-mm skin incision. We observed induced tumour growth as well as impaired wound-healing for all wounds located within 5 mm or less from a tumour, and these wounds were still externally visible 3 weeks after injury (Figure 1). Quantitative analysis of these phenomena will be reported in the following sections.

Histological sections of control incisions showed extensive angiogenesis on the second day after injury (Figure 2A) and complete re-epithelialization by day 7, leaving a thickened fibrotic scar 14 days after injury (Figure 2B). Similar kinetics of healing was observed for incisions located further than 5 mm from a tumour. On the other hand, wounds located within less than 5 mm of tumours did not complete re-epithelialization even by 3 weeks,

© Cancer Research Campaign 1998

**Figure 4** MRI gradient echo images of full-thickness dermal incisions in nude mice. Gradient echo images were obtained on a Bruker 4.7 T Biospec (TE 10.5 ms, TR 100 ms, in plane resolution 110 μm, slice thickness 0.5–0.6 mm). Incisions are marked by white arrows. (**A**) Upper slice of the outer cell layers of the skin, 1 day after incision. (**B**) Second slice of the inner subcutaneous region of the skin, 1 day after incision. Note the darkening of the periphery of the incision. (**C**) Second slice of the inner subcutaneous region of the skin, 2 days after incision, spheroid implanted at incision site. (**D**) Second slice of the inner subcutaneous region of the skin, 7 days after incision, spheroid implanted at incision site (same mouse as in **C**). (**E**) Second slice of the inner subcutaneous region of the skin, no spheroid, 2 days after incision. Note the darkening of the periphery of the incision. (**F**) Second slice of the inner subcutaneous region of the skin, no spheroid, 7 days after incision (same mouse as in **E**). Note the inhibition of vessel regression and wound-healing in tumour wounds (**D**) relative to normal incisions (**E** and **F**). Blood clotting is apparent in the first 2 days after injury (**C** and **E**, black arrows)

and inflammatory cells were observed between the wound and the tumour (Figure 2C). The tumours were significantly more invasive in these cases and infiltrated the dermis (Figure 2C). Tumours located further than 5 mm from the injury grew to smaller size and remained encapsulated at 3 weeks after implantation (Figure 2D). Even when these tumours reached a similar size, they remained encapsulated and did not invade the dermis or disturb the integrity of the epithelium (data not shown).

### Tumour angiogenesis is enhanced by tissue injury

The spatial dependence and temporal kinetics of tumour implantation were evaluated using MRI. When spheroids were implanted precisely at the incision site, the lag in tumour growth was shortened to 2.9 ± 0.2 ($n = 3$), relative to a 5.15 ± 1.6 ($n = 5$) day lag observed for spheroids implanted 5 mm or more from the incision (Abramovitch et al, 1995). Accordingly, tumour diameter, 3 weeks after implantation, was 3.78 ± 0.58 mm ($n = 8$) for spheroids implanted at the site of the incision, relative to 2.16 ± 0.24 mm ($n = 6$) measured for spheroids implanted far from the incision ($P = 0.0001$, Student's $t$-test). This correlated with the enhanced vascularization around the spheroid measured after 2–3 days, which was significantly larger than that observed for spheroids implanted further than 5 mm from the incision (Figure 3A). AVD measured by MRI 9 days after implantation was 2.2-fold larger for spheroids implanted on the incision relative to tumours implanted further away ($P = 0.013$). Spheroids implanted 5 mm or more from the incision showed no detectable enhancement in the rate of vascularization or progression.

© Cancer Research Campaign 1998

**Figure 5** Growth of vascular tumours can be enhanced by incisions. Spheroids were implanted 1 cm away from the incision. Second incisions were made 11 days after implantation at 0.1 and 0.8 cm from the tumour. Note the enhancement observed in tumour growth as a result of the second incision in the following specific cases. Immediate acceleration of tumour growth was observed for tumours located within 5 mm from the injury (O). More distant injuries (> 0.8 cm) accelerated tumour growth only when the incision damaged the existing tumor vasculature (△). Distant injuries that did not perturb tumour vasculature (□) had no effect, and tumours progressed as in control mice (●). -O-, 0.1 cm; -△-, 0.8 cm (vessel damage); -□-, 0.8 cm, (no vessel damage); -●-, no second incision

### Perturbation of wound angiogenesis in the presence of a tumour

Wound-healing was followed using gradient echo images obtained from two consecutive slices of 0.6 mm thickness (Figures 3B and 4). The first slice covered the outer cell layers of the skin, including the epidermis and part of the dermis (Figure 4A). The second slice covered the inner cell layers of the skin dermis and the subcutaneous blood vessels (Figure 4B). The incision was clearly visible in both slices at the first day after injury (Figures 4A and B). Blood clotting was apparent during the first 2 days after injury, primarily in the first slice but occasionally also in the inner slice (Figure 4A, C and E). Such clots showed features characteristic of susceptibility artifacts and complete loss of signal in their centre (Posse and Aue, 1990). These blood clots disappeared usually by day 3 after injury and were visually different and distinguishable from the gradual darkening of the 1-mm periphery of angiogenic stimuli (wounds, tumours or beads containing angiogenic growth factors), which correlated with neovascularization.

Images of the second slice showed initial brightening of the wound that was most apparent on days 1–2 after surgery and was related to fluid accumulation associated with inflammation (Figure 4B and E). This was followed by progressive darkening of the periphery of the incision that correlated with intense neovascularization and was maximal on days 3–4 from injury (Figure 3B, 'infinity'). At later stages of healing, the darkening of the wound periphery in gradient echo images decreased and, accordingly, blood vessels disappeared from the scar region. At its maximum, signal loss around the incision was about twofold larger than that observed for the steady-state angiogenic contrast around an implanted C6 rat glioma tumour (Abramovitch et al, 1995). Within 5–6 days, the wound seemed to be fully recovered externally and there was no detectable contrast around the incision in NMR images (Figure 3B and 4F). The remaining scar was slightly hyperintense relative to the background and was devoid of large blood vessels.

While healing of a normal incision was completed within 10 days, healing of an incision in the presence of a tumour was impaired. The spatial geometrical constraints of this effect were studied on spheroids implanted at different distances from the incision (Figure 3B). When the spheroid was implanted in the site of incision, the wound was externally apparent even after 18 days (Figure 1). For these mice, angiogenic contrast around the incision did not disappear, and the apparent vessel density (AVD) in the periphery of the incision remained high (Figures 3B and 4D). When the spheroid was implanted further than 0.5 cm from the incision, there was no visible interference with the healing process. Sham implantation of agarose beads of a similar diameter (1–5 mm), at different distances from the incision, did not inhibit wound-healing ($n$ = ten mice; two incisions in each mouse).

### Tissue injury induces the progression of vascular tumours

The induction of tumour growth observed for tumours located within 5 mm of injury was also apparent when incisions were applied on already vascularized tumours. In each mouse, a single tumour was initiated from implanted spheroid as reported previously (Abramovitch et al, 1995). A 4-mm incision was made 11 days after spheroid implantation. When the incision was very close to the spheroid (0.1 cm), tumour growth was immediately enhanced, with no apparent lag (Figure 5). In this case, tumour size 7 days after performing the incision was twofold larger than with no incision [7.45 ± 0.18 $mm^3$ ($n$ = 2) and 3.57 ± 0.46 $mm^3$ ($n$ = 5) respectively; $P < 0.05$, Student's $t$-test]. Incisions that were further than 5 mm from the tumour promoted tumour growth only when they caused partial disruption of the tumour perfusion. In such cases, enhancement of tumour growth was observed after a lag of about 5 days (Figure 5), and massive vascularization was observed on the side of the tumour facing the incision, originally perfused by vessels damaged by the incision (Figure 6). Tumour-doubling time, 7 days after the incision, was the same for both proximal and distant incisions, which disrupted tumour perfusion, and was threefold higher than for mice with no incision or with an incision that did not perturb tumour perfusion (95% significance level, ANOVA) (Figure 5).

### DISCUSSION

The increased probability of recurrence and accelerated tumour growth in the location of the tissue injury are common clinical complications associated with the invasive procedures frequently used in cancer therapy, including biopsy and surgery. In the study reported here, we applied quantitative MRI to follow angiogenesis and tumour growth in tumour–wound systems of C6 glioma in nude mice. The assumption that underlies this study is that angiogenesis is limiting the rate of growth of an implanted avascular multicellular tumour spheroid and that the bottle neck is the limited production of angiogenic growth factors rather than a limited capacity of the host to respond to the angiogenic stimuli. In such a case, increasing the angiogenic stimulation will also increase the extent of vascularization and subsequently the initial rate of tumour growth.

 © Cancer Research Campaign 1998

**Figure 6** Tumour neovascularization induced by distant incision. Spheroid (arrow head) was implanted 1 cm away from the incision. A second 4-mm incision (arrows) was made 9 days (**A** and **B**) or 11 days (**C** and **D**) after spheroid implantation at 0.8 cm from the tumour. Note the massive vascularization on the left side of the tumour facing the incision, which damaged the tumour vasculature, in comparison to the other side of the tumour (**A** and **B**). Neovascularization appears as redness in photographs (**A**) and as darkening in MRI images (**B**). When the incision did not damage the vasculature, no effect was observed on tumour neovascularization (**C** and **D**) and on tumour growth (Figure 5). (**A** and **C**) Skin photographs taken 13 days after the second incision. (**B** and **D**) Gradient echo images of the inner region of the skin from the same mouse obtained 7 days after the second incision. Black bars are 2 mm

Skin injuries are associated with extensive angiogenesis during the first 3 days of healing and thus could provide an angiogenic stimulus that will be stimulatory to tumour implantation. In accordance with the supposition laid above, we find that injury in proximity to a tumour invokes an angiogenic activity that is larger than that provided by either one of them alone. These results imply that the angiogenic capacity of the vascular system is not saturated by each of these triggers. Moreover, these results suggest that any genetic transformation or environmental milieu that will increase the production of angiogenic stimuli could also increase neovascularization and promote tumour growth.

We have previously demonstrated that after vascularization of implanted C6 glioma spheroids the expression of VEGF decreases to the residual low level of constitutive expression (Shweiki et al, 1995). Correspondingly, we found that the rate of tumour growth decreases following Gompertz rather than exponential kinetics (Abramovitch et al, 1995). One possible explanation for these results is that the rate of tumour progression 1–2 weeks after spheroid implantation is limited by the reduced expression of VEGF. By invoking hypoxic stress in the tumour, we would expect to get a second wave of neovascularization and accelerated growth. Indeed, when a second distant (> 8 mm) incision was created 11 days after tumour implantation, damaging vessels irrigating the tumour, we observed a latent enhancement of tumour growth. Such enhancement was not observed in cases in which a similar incision did not damage vessels irrigating the tumour. This effect of a distant (> 8 mm) incision on a vascular tumour was observed after a lag of approximately 4–5 days and was associated with distinct neovascularization of the side of the tumour facing the incision, which was larger than the vascularization on the other side of either the incision or the tumour. These findings are consistent with the hypothesis that by making the tumour partly hypoxic, the levels of stress-induced angiogenic growth factors (such as VEGF) increase in the tumour, leading to a wave of neovascularization that could support a burst of regrowth.

Another important issue that appeared in the course of these experiments was the observation that wounds located on tumours did not completely heal even after 3 weeks, whereas normally the scar would be almost undetectable externally by 7 days. This observation reproduces a previous report that tumours, regardless of cell type, inhibit wound-healing (Gatenby and Taylor, 1990). Such inhibition of healing of the wound by a tumour could theoretically imply a continuous release of growth factors from the wound further stimulating tumour progression, resulting in a vicious cycle in which accelerated tumour progression is driven by persistent non-healing wounds.

We report here the obstruction of vascular regression in wounds located in proximity to tumours. We would like to postulate that, in normal healing, restoration of perfusion and normoxia down-regulates the production of angiogenic factors (e.g. VEGF), and this will lead to vessel regression. Indeed, it was recently shown that VEGF acts as a survival factor for newly formed capillaries, and VEGF withdrawal results in vessel regression (Alon et al, 1995). In the presence of a proximal C6 glioma spheroid, VEGF released by the spheroid (Shweiki et al, 1995) may be sufficient to confer survival of the wound vasculature and prevent vessel regression. Angiogenesis is generally believed to be an essential component of healing during the first days after injury. In view of the data presented here, possibly at later stages after injury, vascular regression is obligatory for completion of the healing, and healing may be blocked by the persistent neovascularization.

MRI as a tool for studying angiogenesis provides a major advantage over histology in the ability to monitor a full-time course non-invasively. The commonly used in vivo assays for angiogenesis, such as the window chambers, involve massive injury and limited access to the tumour and thus cannot be used for monitoring the effects of a defined injury on tumour vascularization. Finally, MRI can potentially be applied for clinical assessment of tumour involvement during recovery from surgery.

In summary, we showed here that proximal wounds promote angiogenesis and growth of avascular tumours. In our model, which uses small, full-thickness, dermal-incisional wounds, this mechanism requires close proximity of the injury to the tumour and was found to be spatially restricted to less than 5 mm. The spatial restriction of this effect will probably vary between tissues depending on the rates of diffusion and clearance of the different mitogens. In addition, wounds damaging the tumour vasculature promoted tumour growth, even when the distance between the tumour and the injury was greater than 5 mm, suggesting that such injuries invoke stress-induced angiogenesis in the tumour. In view of the elevated angiogenic activity in the wound–tumour systems and the correlation between impaired healing and inhibition of vessel regression, it would be interesting to evaluate the effect of anti-neovascularization therapy, which would, on one hand, inhibit tumour angiogenesis and, on the other hand, would impose regression of vessels around the persistent wound. Finally, non-invasive kinetic MRI analysis of the angiogenic response after dermal incision provided a unique sensitivity to the accelerated neovascularization associated with the presence of proximal tumours, which could potentially be induced by the incision, leading to local tumour recurrence. The diagnostic potential of MRI in early detection of tumour involvement in wounds should be evaluated.

### ACKNOWLEDGEMENTS

This work was supported by a Research Career Development Award from the Israel Cancer Research Fund and a research grant from the US-Israel Binational Science Foundation BSF 93-00073 (to MN). MN is an incumbent of the Dr Phil Gold Career Development Chair in Cancer Research. RA is a recipient of a fellowship from the Charles Clore foundation. We would like to thank Ms Dorit Natan and Dr Alon Harmelin for their help in preparation and analysis of the histological sections.

### REFERENCES

Abramovitch R, Meir G and Neeman M (1995) Neovascularization induced growth of implanted C6 glioma multicellular spheroids: magnetic resonance microimaging. *Cancer Res* **55**: 1956–1962

Abramovitch R, Frenkiel D, Meir G, Hellerqvist C-G and Neeman M (1997) Mapping neovascularization and anti-neovascularization therapy: correlation between NMR and light microscopy. In *Proceedings of the 5th ISMRM*, Vancouver, Canada, 12–18 April 1997, p. 490.

Alon T, Hemo I, Itin A, Pe'er J, Stone J and Keshet E (1995) Vascular endothelial growth factor acts as a survival factor for newly formed retinal vessels and has implications for retinopathy of prematurity. *Nature Med* **1**: 1024–1028

Deelman HT (1927) The part played by injury and repair in the development of cancer. *Br Med J* **1**: 872

Detmar M, Yeo KT, Nagy JA, Van de Water L, Brown LF, Berse B, Elicker BM, Ledbetter S and Dvorak HF (1995) Keratinocyte-derived vascular permeability factor (vascular endothelial growth factor) is a potent mitogen for dermal microvascular endothelial cells. *J Invest Dermatol* **105**: 44–50

Dvorak HF (1986) Tumors: wounds that do not heal. Similarities between tumour stroma generation and wound healing. *N Engl J Med* **315**: 1650–1659

Dvorak HF, Harvey VS, Estrella P, Brown LF, McDonagh J and Dvorak AM (1987) Fibrin containing gels induce angiogenesis. Implications for tumor stroma generation and wound healing. *Lab Invest* **57**: 673–686

Fisher B, Fisher ER and Feduska N (1967) Trauma and the localization of tumor cells. *Cancer* **20**: 23–30

Folkman J (1995) Angiogenesis in cancer, vascular, rheumatoid and other disease. *Nature Med* **1**: 27–31

Folkman J and Shing Y (1992) Angiogenesis. *J Biol Chem* **267**: 10931–10934

Frank S, Hubner G, Breier G, Longaker MT, Greenhalgh DG and Werner S (1995) Regulation of vascular endothelial growth factor expression in cultured keratinocytes. Implications for normal and impaired wound healing. *J Biol Chem* **270**: 12607–12613

Gatenby RA and Taylor DD (1990) Suppression of wound healing in tumour bearing animals as a model for tumour–host interaction: mechanism of suppression. *Cancer Res* **50**: 7997–8001

Giray CB, Sungur A, Atasever A and Araz K (1995) Comparison of silk sutures and n-butyl-2-cyanoacrylate on the healing of skin wounds. A pilot study. *Aust Dent J* **40**: 43–45 issn: 0045–0421

Green MM, Boudreau N and Bissell MJ (1994) Inflammation is responsible for the development of wound-induced tumors in chickens infected with rous sarcoma virus. *Cancer Res* **54**: 4334–4341

Haran-Ghera N, Trainin N, Fiore-Donati L and Berenblum I (1962). A possible two-stage mechanism in rhabdomyosarcoma induction in rats. *Br J Cancer* **16**: 653–664

Lynch SE (1991) Interactions of growth factors in tissue repair. *Prog Clin Biol Res* **365**: 341–357

Moulin V (1995) Growth factors in skin wound healing. *Eur J Cell Biol* **68**: 1–7

Murthy SM, Goldschmidt RA, Rao LN, Ammirati M, Buchmann T and Scanlon EF (1989) The influence of surgical trauma on experimental metastasis. *Cancer* **64**: 2035–2044

Murthy MS, Summaria LJ, Miller RJ, Wyse TB, Goldschmidt RA and Scanlon EF (1991) Inhibition of tumor implantation at sites of trauma by plasminogen activators. *Cancer* **68**: 1724–1730

Ogawa S and Lee TM (1990) Magnetic resonance imaging of blood vessels at high fields: in vivo and in vitro measurements and image simulation. *Magn Reson Med* **16**: 9–18

Orr FW, Adamson IY and Young L (1986) Promotion of pulmonary metastasis in mice by bleomycin-induced endothelial injury. *Cancer Res* **46**: 891–897

Paschkis K, Cantarow A, Stasney J and Hobbs JH (1955) Tumor growth in partially hepatectomized rats. *Cancer Res* **15**: 579–582

Posse S & Aue WP (1990) Susceptibility artifacts in spin-echo and gradient echo imaging. *J Magn Reson* **88**: 473–492

Rak JW, Hegmann EJ, Lu C and Kerbel RS (1994) Progressive loss of sensitivity to endothelium-derived growth inhibitors expressed by human melanoma cells during disease progression. *J Cell Physiol* **159**: 245–255

© Cancer Research Campaign 1998

Schackert HK and Fidler IJ (1989) Development of an animal model to study the biology of recurrent colorectal cancer originating from mesenteric lymph system metastases. *Int J Cancer* **44**: 177–181

Schiffenbauer YS, Tempel C, Abramovitch R, Meir G and Neeman M (1995) Cyclocreatine accumulation leads to cellular swelling in C6 glioma multicellular spheroids: diffusion and one-dimensional chemical shift nuclear magnetic resonance microscopy. *Cancer Res* **55**: 153–158

Shweiki D, Neeman M, Itin A and Keshet E (1995) Induction of vascular endothelial growth factor expression by hypoxia and by glucose deficiency in multicell spheroids: implications for tumor angiogenesis. *Proc Natl Acad Sci USA* **92**: 768–772

Sugarbaker EV, Ketcham AS and Cohen AM (1971) Studies of dormant tumor cells. *Cancer* **28**: 545–552

Sutherland RM (1988) Cell and environment interactions in tumor microregions: the multicell spheroid model. *Science* **240**: 177–184

Waleh NS, Brody MD, Knapp MA, Mendonca HL, Lord EM, Koch CJ, Laderoute KR and Sutherland RM (1995) Mapping of the vascular endothelial growth factor-producing hypoxic cells in multicellular tumor spheroids using a hypoxia-specific marker. *Cancer Res* **55**: 6222–6226

© Cancer Research Campaign 1998